# Idiopathic Paracentral Acute Middle Maculopathy in a Healthy Pregnant Woman: A Case Report

**DOI:** 10.1155/crop/1925710

**Published:** 2026-04-02

**Authors:** K. Broniarek, K. Michalska-Małecka

**Affiliations:** ^1^ Department of Ophthalmology, Medical University of Gdansk, Gdansk, Poland, mug.edu.pl

**Keywords:** idiopathic, OCT, OCTA, PAMM, pregnancy

## Abstract

**Purpose:**

The purpose of this study is to report a case of idiopathic paracentral acute middle maculopathy (PAMM) in a healthy woman during the first trimester of an uncomplicated pregnancy.

**Observations:**

A 28‐year‐old woman at 14 weeks gestation presented with sudden onset of a paracentral scotoma in her right eye. Although her best‐corrected visual acuity remained 20/20 OU, dilated fundus examination, optical coherence tomography (OCT), and OCT angiography (OCTA) revealed paracentral acute middle maculopathy. A comprehensive systemic workup returned normal results, suggesting no underlying general condition. Over 1 year of follow‐up, resolution of fundus and OCT findings, a gradual increase in deep‐plexus vessel density in the affected area on OCTA was observed, and improvement in the patient′s visual field was noted.

**Conclusions and Importance:**

Despite its low incidence, PAMM should be included in the differential diagnosis of acute visual disturbances in pregnant women—even when no other risk factors are present. OCTA can be a valuable modality for both diagnosis and monitoring of PAMM.

## 1. Introduction

Paracentral acute middle maculopathy (PAMM) is an eye condition first described by Sarraf et al. in 2013 as a variant of acute macular neuropathy (AMN) [1]. On optical coherence tomography (OCT), PAMM appears as a hyperreflective band in the inner nuclear layer (INL), which later progresses to INL thinning and atrophy. Unlike AMN—where disruption of the interdigitation zone (IZ) and ellipsoid zone (EZ) is often seen—PAMM typically preserves the IZ/EZ complex. PAMM is believed to result from microvascular hypoperfusion predominantly affecting the deep capillary plexus, a finding that can be visualized on optical coherence tomography angiography (OCTA) [2, 3].

Although multiple risk factors—chiefly hypercoagulable states—have been implicated in PAMM pathogenesis, the underlying mechanisms remain incompletely understood. Notably, pregnancy itself represents a prothrombotic state. Existing reports describe cases of PAMM in pregnant patients, most of whom had additional identifiable risk factors. We report a unique case of idiopathic PAMM occurring during an uncomplicated pregnancy in a healthy woman.

## 2. Case Presentation

A 28‐year‐old Caucasian woman at 14 weeks of unremarkable pregnancy presented with a 1‐day history of a unilateral paracentral scotoma in the right eye. She described an acute, arrowhead‐shaped defect in the lower visual field without ocular pain, which developed upon awakening. She denied any other systemic symptoms. Her past ophthalmological history was unremarkable. The patient had been treated for migraines with auras and at first attributed the visual disturbances to migraine prodrome. The persistence of visual symptoms without headache prompted her to seek evaluation in the emergency department.

She denied recent eye trauma or use of psychoactive substances, including caffeine, alcohol, and nicotine. She was not taking any medications; no vaccinations were administered to her in the months following this event. It was her first gestation, and no related complications had been diagnosed by the obstetrician overseeing her pregnancy, including eclampsia/preeclampsia. She had no history of miscarriage. She had a history of migraine attacks, but no migraine‐related symptoms were present before, during, or after the diagnostic period. Carotid Doppler ultrasound, echocardiography and neurological work‐ up revealed no pathologies.

On ophthalmological evaluation, best‐corrected visual acuity was 20/20 (Snellen) in both eyes. Intraocular pressure was 16 mmHg in the right eye and 17 mmHg in the left eye. Direct and indirect pupillary responses were normal, and color vision testing (Ishihara test) showed no signs of dyschromatopsia.

**Figure 1:**
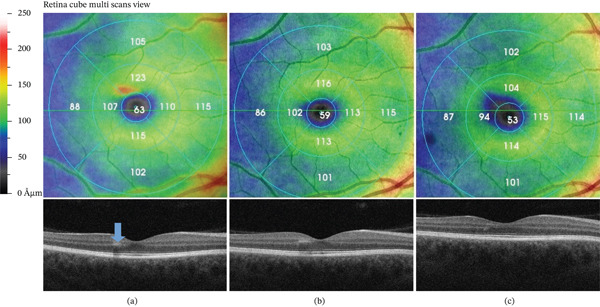
Hyperreflective, band‐like focal lesion in the inner nuclear layer of the superior parafovea (blue arrow) visible on the day of diagnosis. Interdigitation zone (IZ) and ellipsoid zone (EZ) disruptions were not identified (a). Further resolution of the hyperreflective band, with progressive INL atrophy and thinning, is visible at 3 months (b) and 1 year (c) after diagnosis.

**Figure 2:**
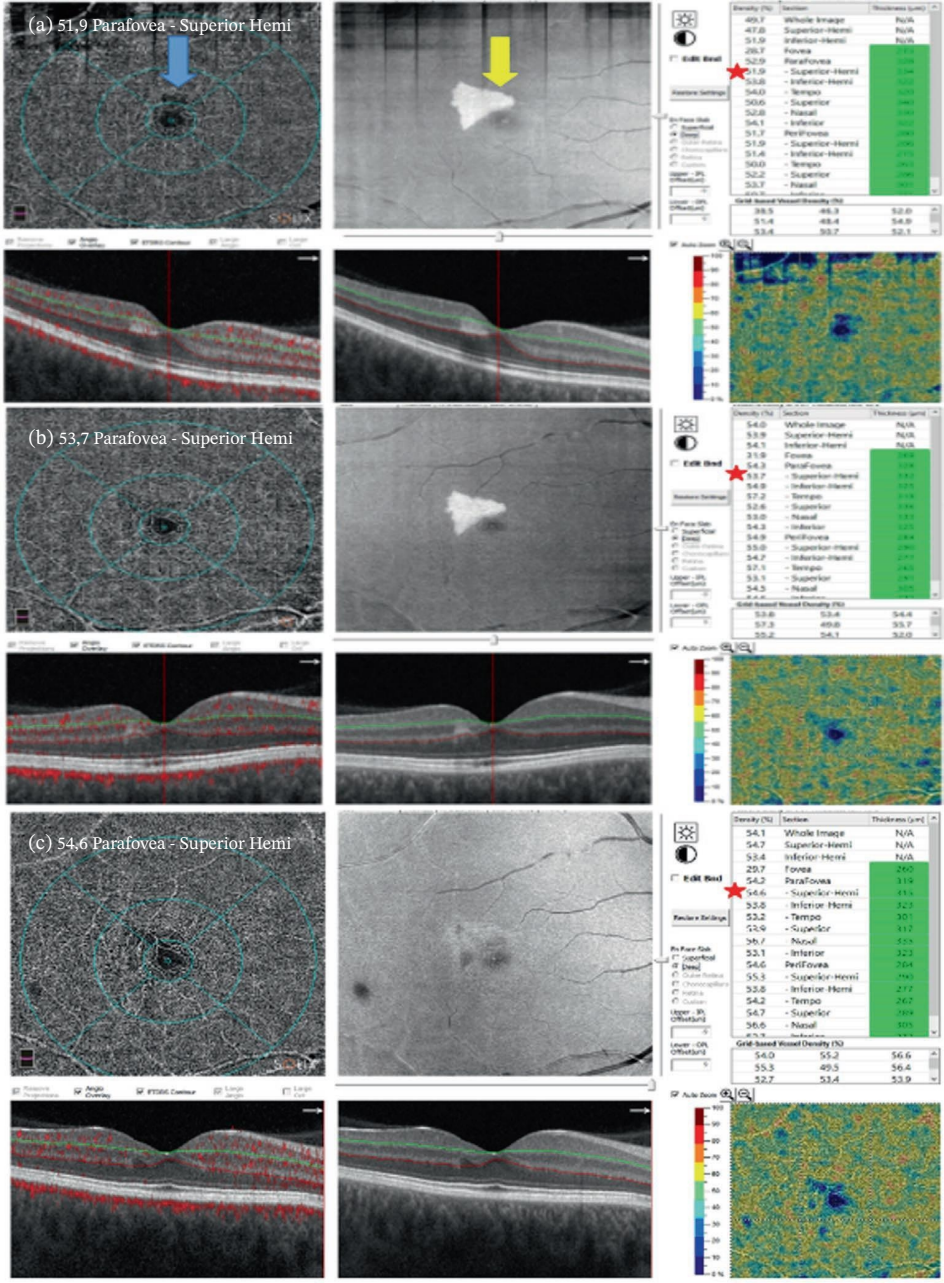
OCTA scans reveal capillary dropout in the affected area (blue arrow). Corresponding OCT En Face images show a hyperreflective zone (yellow arrow). Tables show a gradual increase of vessel density in the deep plexus of superior parafovea (red star). Images correspond to day of diagnosis (a), 3 months after diagnosis (b), and 1 year after diagnosis (c).

Dilated fundus examination of the right eye revealed grayish mottling in the upper paravoeal region; the left fundus was unremarkable. Hyperreflective band‐like focal lesion in the INL of the superior parafovea was noted in OCT (Figure 1). In the corresponding region, OCTA showed perfusion deficit in the deep retinal plexus measured in reduced vessel density (Figure 2). Fluorescein angiography performed postpartum showed no abnormalities in the described area.

Conducted blood investigations, including a hypercoagulability panel (protein C and S, activated protein C resistance, and dRVVT), fasting glucose levels, C‐reactive protein, and blood count were normal. Blood pressure was within normal values.

The pregnancy was uneventful and culminated in the delivery of a healthy infant at term.

## 3. Management and Follow‐Up

Since no other pathologies were identified, the patient was observed regularly with OCT and OCTA scans at every visit. Vessel density was analyzed using the automatic AngioVue software. No treatment was administered. After 3 months, the patient reported gradual brightening of the visual field defect, which corresponded with an increase in vessel density in deep‐plexus vessel density and a reduction of lesion reflectivity. After 1 year, OCT showed thinning of the affected area and INL atrophy, whereas vessel density continued to increase. Throughout follow‐up, the patient maintained 20/20 vision and at 1 year had no residual scotoma.

## 4. Discussion

PAMM is a rare ocular disease whose etiology is not fully understood. First described by Sarraf et al. in 2013 as Type 1 AMN, its diagnosis is based on OCT scans presenting a characteristic hyperreflective band in the INL region of the macula, which later progresses to INL thinning and atrophy [1]. Gray lesions on fundus examination may be subtle or not visible [1, 2]. Acute‐onset paracentral scotomas can occur yet remain unnoticed by the patient [4, 5]. PAMM can result from various infectious, inflammatory, vascular, or toxic factors leading to occlusion of the capillary plexus surrounding the INL [2]. Many risk factors have been described, for example: eye compression injury causing global ocular ischemia; sickle cell crisis; inflammatory occlusive retinal vasculitis; Purtscher′s retinopathy; postvaccination inflammatory response; giant cell arteritis; diabetic retinopathy; hypertensive retinopathy; migraine disorder; postupper respiratory infection; cardiovascular interventions; and iron deficiency anemia [6–12]. Idiopathic PAMM in healthy individuals has also been reported [13–15].

Several cases of PAMM occurrence have been reported during pregnancy. Pregnancy is a known hypercoagulable state [16–18]. Chen et al. described PAMM in a healthy pregnant woman [19]. Coulon et al. reported a 38‐year‐old pregnant patient with hypercoagulability caused by elevated Factor VIII activity [20]. Hellier et al. described a pregnant woman with a history of migraines and elevated antinuclear titers during hormone therapy after in vitro fertilization [21]. Importantly, in all of the above‐mentioned cases, including ours, disease onset occurred during the first trimester of pregnancy. Jürgens et al. presented a case of a 32‐year‐old patient at 16 weeks′ gestation developing PAMM, in whom cardiologic evaluation revealed a patent foramen ovale [22]. Pecen et al. reported PAMM in pregnancy associated with anemia and hypertension [23]. In our patient, no cardiovascular or hematologic disorders were identified; her only systemic disorder was a history of migraine with visual aura.

Migraine is a complex neurological disorder, typically defined by hemicranial pain, nausea, photophobia, and emesis. Although its pathogenesis remains incompletely understood, studies suggest dysregulated neural activity affecting neuronal signaling, neurochemical balance, and cerebral vasculature. An association between migraine and PAMM has been suggested by several authors, some describing PAMM occurrence following a migraine attack [24–26]. Da Silva et al. even described PAMM co‐occurring after a first migraine attack in pregnancy [27]. Apart from an embolic etiology, PAMM could be caused by vasospasm [3, 28] similar to the stroke‐related mechanisms described in migraine [29]. It is known that migraine can influence retinal circulation, as it is associated, for example, with increased risk of a retinal artery occlusion [30]. Pang et al. gathered evidence in a meta‐analysis showing that patients with migraine have reduced vessel density in both the superficial and deep capillary plexuses of the macula on OCTA [31], confirming retinal blood flow restriction. In our case, no migraine attack was documented at the time of PAMM onset. Thus, an association between the patient′s migraine history and the development of PAMM cannot be established. Nonetheless, similarity in underlying mechanisms of mentioned diseases‐particularly transient retinal microvascular vasospasm is noticeable. Similar to our patient′s case, PAMM has also been reported in pregnant individuals with migraine, even in the absence of an attack at presentation [32]. Unlike our patient, that case involved a preexisting cardiovascular risk factor: arterial hypertension. PAMM, as an ischemic retinal event, could potentially occur more frequently in patients with migraine, but large studies have not yet been conducted.

No proven treatment exists for PAMM; therefore, careful observation was pursued in our case. For follow‐up examinations, OCTA scans were used because they present no risks related to intravenous contrast, unlike the gold standard—fluorescein angiography. Follow‐up OCTA measurements showed a gradual increase in deep plexus vessel density and have been observed in other PAMM cases as well, suggesting reperfusion or revascularization of the affected area [33]. Thorough evaluation for additional risk factors contributing to PAMM development was conducted. Early diagnosis of PAMM could be the first sign of more serious ocular events—such as central retinal vein occlusion, central retinal artery occlusion, or macular ischemia—to come [34, 35]. In every case of PAMM, cardiovascular risk should be carefully assessed to rule out life‐threatening conditions [36].

Our case documents a rare occurrence of idiopathic PAMM in the first trimester of an uncomplicated pregnancy. The limited scientific data preclude establishing a definitive link between migraine and PAMM development in this patient. Further prospective research is required to estimate frequency and assess mechanisms of PAMM in pregnancy.

## Funding

No funding was received for this manuscript.

## Ethics Statement

Institutional review board approval was not required for this study, in accordance with the local guidelines.

## Consent

Written consent has been obtained from the patient.

## Conflicts of Interest

The authors declare no conflicts of interest.

## Data Availability

The data that support the findings of this study are available from the corresponding authors upon reasonable request.
